# Assessing mental health literacy of primary health care workers in Kenya: a cross-sectional survey

**DOI:** 10.1186/s13033-021-00481-z

**Published:** 2021-06-01

**Authors:** Elijah Marangu, Fethi Mansouri, Natisha Sands, David Ndetei, Peterson Muriithi, Karen Wynter, Helen Rawson

**Affiliations:** 1 National Indigenous Knowledges Education Research Innovation (NIKERI) Institute, Deakin University, 75 Pigdons Road, Waurn Ponds, VIC 3216 Australia; 2 Institute for Citizenship & Globalisation, Deakin University, Burwood Campus, 221 Burwood Highway, Burwood, VIC 3125 Australia; 3 Nurse Talk PTY, Melbourne, Australia; 4 Department of Psychiatry, Nairobi University, P.O Box 48423-00100, Nairobi, Kenya; 5 School of Population Health, Nairobi University, P.O Box 19676-00202 KNH, Nairobi, Kenya; 6 School of Nursing & Midwifery, Deakin University, Burwood Campus. Building Y, 221 Burwood Highway, Burwood, VIC 3125 Australia; 7 Nursing & Midwifery, Monash University, Level 3, Building 13D. 35 Rainforest Walk, Clayton, VIC 3800 Australia

**Keywords:** Capacity building, Mental health care, Primary health care, Mental health literacy, Low and middle-income countries

## Abstract

**Aim:**

To assess mental health literacy of health workers in primary health care services in Kenya.

**Background:**

Mental illness is common in Kenya, yet there are fewer than 500 specialist mental health workers to serve Kenya’s population of over 50 million. The World Health Organization recommends the integration of mental health care into primary health care services to improve access to and equity of this care, especially in low and middle-income countries. An important step to integrating mental health care into primary health care services is to determine mental health literacy levels of the primary health care workforce.

**Method:**

A cross-sectional survey using Jorm’s Mental Health Literacy Instrument (adapted for the Kenyan context) was administered to 310 primary health care workers in four counties of Kenya.

**Results:**

Of the 310 questionnaires distributed, 212 (68.3%) were returned. Of the respondents, 13% had a formal mental health qualification, while only 8.7% had received relevant continuing professional development in the five years preceding the survey. Just over one third (35.6%) of primary health care workers could correctly identify depression, with even fewer recognising schizophrenia (15.7%).

**Conclusions:**

This study provides preliminary information about mental health literacy among primary health care workers in Kenya. The majority of respondents had low mental health literacy as indicated by their inability to identify common mental disorders. While identifying gaps in primary health care workers’ mental health knowledge, these data highlight opportunities for capacity building that can enhance mental health care in Kenya and similar low and middle-income countries.

## Background

Mental health care for people living in low and middle-income countries (LMICs) has been described as inadequate, inefficient and inequitable [1, 2]. This leads to a ‘treatment gap’, which is defined as the number of people with a mental disorder that do not receive mental health care [3–5]. The treatment gap in most LMICs is estimated to be as high as 85% [6]. In 2002, the World Health Organization (WHO) launched the Mental Health Global Action Program (commonly referred to as mhGAP), in response to the increasing disease burden attributed to mental, neurological and substance use disorders, especially in LMICs [7]. A key strategy of the mhGAP was integration of mental health services into primary health care settings to ensure accessible, affordable and equitable mental health care [2, 8, 9]. Successful implementation of strategies such as mhGAP in LMICs can be enhanced by adopting Amartya Sen’s Capability Approach [10, 11]. Capability Approach involves thorough analysis of context and affirming existing health infrastructure and workforce to improve health outcomes [10]. Focusing on Kenya, this study utilised Jorm’s Mental Health Literacy survey tool to describe mental health knowledge and attitudes of the primary health care workers in four counties of Kenya.

Kenya is a low-income country in East Africa with a population of over 50 million people [12]. Overall, Kenya is a resource-poor country that faces significant challenges to provide health services to its population, especially those living in rural and remote areas [13]. It has an aggregate medical personnel to population ratio of 13:10,000, which is far below the minimum of 41:10,000 set by the International Labour Organisation as being adequate to achieve Sustainable Development Goals in LMICs [14]. This shortage of medical personnel is reflected in most of the health sector, but especially in mental health care where the specialist mental health workforce consists of only 116 psychiatrists and less than 500 registered psychiatric nurses [15, 16]. Additional constraints to mental health care provision in Kenya include lack of adequate financing, poor health infrastructure, limited therapeutic resources and socio-cultural stigma [17, 18]. Recent research by Mutiso et al. (2020) using the WHO Assessment Instrument for Mental Health Systems (WHO-AIMS) has pointed to lack of administrative structures for mental health care provision such as policies and governance. This has resulted to low prioritisation of mental health care in Kenya [19].

### Kenya’s health system: structure and key components

**Fig. 1:**
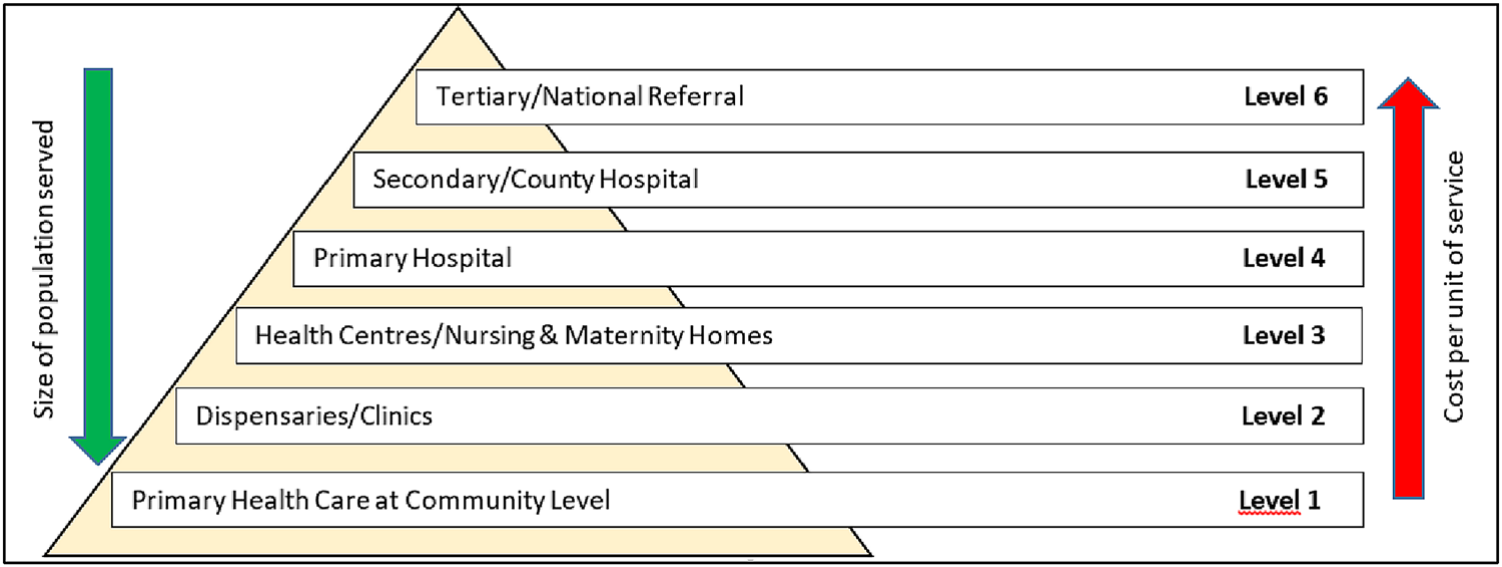
Structure of Kenya’s health system

Under the Kenya Health Policy 2014–2030, a six-level structure of the health system is outlined (Fig. 1), beginning with very basic primary health care at community level (level 1) to tertiary care (level 6) [20]. Primary health care services are provided in levels 1 to level 4 of the Kenyan health system [13]. It has been acknowledged that primary health care services in Kenya are the main, and often the only, available source of health care for the majority of Kenyans, yet, the highest percentage of the health budget is concentrated on tertiary services that serve a much smaller population [21, 22], as illustrated in Fig. 1.

### Mental health care in Kenya

Kenya has no record of national surveillance data on mental illness. Disease burden related to mental illness for Kenya is difficult to estimate because of lack of resources and governance systems that would make disease surveillance possible [23]. The *Mental Health Atlas* series published by WHO provides the most comprehensive surveillance data and resources for mental health for affiliate countries and regions globally [24]. The series is particularly important as a key source of information on mental health system and resources for low and middle-income countries. Currently, the *Mental Health Atlas* country profile for Kenya provides in-patient data generated by three psychiatric hospitals. The *Mental Health Atlas* reports no information for community care or residential care, or prevalence rates for mental illness in Kenya [24]. Previous studies undertaken in parts of Kenya have reported high prevalence rates of mental disorders, Jenkins et al*.* reported a 10.8% point prevalence of common mental disorders among the adult population in Maseno, a rural town in Nyanza region of Kenya [30]. Earlier studies have reported even higher neuropsychiatric prevalence rates of 25% for Kenya [17, 25]. Taken together, this indicates an urgent need for a skilled workforce at the primary health care level with the appropriate skills to assess, plan and provide care for people who experience mental illness across Kenya.

The mental health care system in Kenya is centred around three main public level 6 hospitals and a few private providers in major urban centres [17]. Similar to other low-income countries, mental health services in Kenya are not adequately funded, with < 5% of the health budget dedicated to mental health care [19]. A key purpose of tertiary mental health facilities is to manage exacerbations of mental disorders and to control for associated risks. The outcome of tertiary driven mental health services is often expensive institutional care that may be characterised by long term care, human rights abuse, lack of advocacy and stigma [26, 27]. A health system that incorporates mental health services at the primary health care level has a number of advantages, including holistic care and better management of mental health complications related to chronic medical conditions such as cardiovascular disease and diabetes [2]. Additional benefits include improvements in mental health promotion and prevention, enhanced treatment and follow-up, lower costs to patients and their families, and enhanced capacity of the health system to manage mental health problems [3].

### Mental health literacy

The concept of mental health literacy was developed by Anthony Jorm in the late 1990s in Australia [28]; this was in response to the lack of research and action to address the gaps in public knowledge and attitudes towards mental illness. Jorm defined mental health literacy as knowledge and belief about mental disorders which aid their recognition, management and prevention (2000). Therefore, good mental health literacy levels should enable recognition of specific disorders and aid help seeking, knowledge of causes and risk factors, self-treatments, and positive attitudes [28]. Mental health literacy of the primary health care workforce is necessary to ensure successful integration of mental health care into primary health care services. To date, no studies have explored mental health literacy levels of the primary health care workforce in Kenya.

As part of a wider study that assessed mental health service systems in Kenya, this paper focuses on mental health services at primary health care level and reports on the following:

- Mental health literacy, indicated by the capability of primary health care workers to assess, recognise and respond appropriately to symptoms of common mental illnesses in hypothetical characters described in vignettes. For this study, schizophrenia and depression were chosen because these two conditions are associated with high levels of risk and disability and are frequently encountered in clinical settings of Kenya [29, 30];
- Types of support perceived as helpful for people similar to those described in questionnaire clinical vignettes; and
- Attitudes towards people similar to those described in the questionnaire clinical vignettes.

## Methods

In a cross-sectional survey design, a standardised questionnaire was used to assess the mental health literacy of primary health care workers in four counties across Kenya.

### Setting

The setting was 78 primary health care facilities in four counties in central and eastern Kenya. The primary health care facilities comprised level 1 to level 4 of Kenya’s health system. Purposive sampling is frequently used in research to aid matching of the sample to research aims, especially in diverse environments [31]. A purposive sampling strategy was adopted with the aim of collecting data from areas that would typically represent where Kenyans live, these being urban, peri-urban, rural and remote. Nairobi county is very urbanised, Murang’a and Meru county are peri-urban and rural, while Machakos county is peri-urban, rural and remote.

At the time of the study, there were a total of 78 Level 1–4 public health care facilities in the four counties registered with the Kenya Master Facility List [32] at the Ministry of Health (Nairobi). All primary health care workers in Level 1–4 health facilities in these counties were invited to participate.

### Participants

The participants were primary health care workers, including registered nurses, enrolled nurses, clinical officers and doctors. Clinical Officer is a category of middle-level diploma and degree trained health workers who supplement medical services and care that would typically be provided by doctors. Doctors are in short supply in Kenya, Clinical officers and registered nurses are relied upon to assess, diagnose and prescribe treatment for common illnesses in urban, rural and remote parts of Kenya [33].

At the time of data collection there were a total of 5050 primary health care workers employed across the four counties. Of these, approximately 25% (1253) were skilled to work in Level 1–4 primary health care facilities and therefore eligible for this research. A total of 310 participants (approximately 25% of eligible health care workers) participated in the survey. Kenya health workforce data indicate that the majority of these health workers would have been nurses [33, 34].

### The questionnaire

Jorm’s Mental Health Literacy Questionnaire (MHLQ) was identified as the most appropriate survey tool to use in this study because of its reported ability to assess mental health knowledge, attitudes and help-seeking efficacy among health care workers [35]. The 56-item questionnaire assesses knowledge of mental illness including common causes, symptoms and diagnosis, supportive strategies, and exploration of common attitudes towards people who experience mental illness. Jorm’s questionnaire has been widely used to measure mental health literacy levels of health care workers in LMICs such as India [36, 37], Pakistan [38], Nigeria [39] and South Africa [40].

### Questionnaire and clinical vignettes

Jorm’s Questionnaire (including clinical vignettes) was adapted for use in Kenya by two researchers (EM & PM) in this study, and in collaboration with research partners in Kenya prior to data collection, the adapted questionnaire was pilot-tested with 12 health care workers in one mental health facility in Nairobi, Kenya.

Consistent with the two official languages in Kenya, the two vignettes were presented to participants in both English and Swahili versions. The vignettes were only modified for names linguistic and cultural tone, the content of original versions that describe common symptoms for depression and schizophrenia was maintained. The two vignettes are included in Box 1.

**Box 1:**
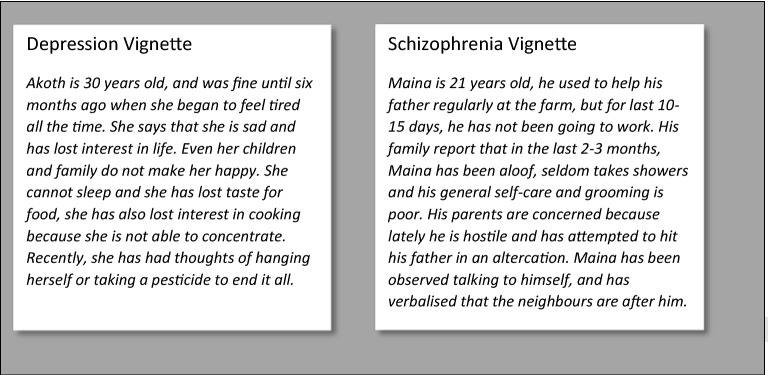
English versions of the depression and schizophrenia vignettes from the questionnaire

### Data collection

Data were collected between June 2014 and January 2015. Due to the unreliability of the postal services in the four counties, the survey packages (containing plain language statements, consent forms and questionnaires) were hand delivered to primary health care facilities in each of the four counties by the first author (EM) and research assistants. The quasi-random sampling approach [41] was used in questionnaire distribution, researcher arranged the questionnaires in batches of 50, consisting of 25 questionnaires with the schizophrenia vignette and 25 with the depression vignette, they were sequentially arranged so that every healthcare worker had an equal chance of getting either version of the questionnaire.

Before handing the questionnaire to the participants, consent was sought via completion and signing of a consent form. The questionnaire took approximately 40 to 50 min to complete. A two-week period was stipulated for the completion of the questionnaires, after which they were collected by the first author (EM) and research assistants. A total of 310 questionnaires were distributed to primary health care workers across the four counties.

### Data analysis

IBM SPSS Statistics [42] was used to analyse quantitative data. Descriptive statistics are presented for socio-demographic characteristics as well as for responses to the questions related to the vignettes. Associations between accuracy of diagnoses for the person in the vignette and various socio-demographic characteristics were analysed using Chi-square tests (χ^2^) where p-value was set at 0.05.

## Results

### Demographic characteristics

The social and demographic characteristics of participants are summarised in Table 1. Of the 310 questionnaires distributed, 212 completed questionnaires were returned, yielding a 68.3% response rate. The 212 primary health care workers ranged in age from 24 to 62 years, with a mean age of 32 years. The majority were female (76%), under 40 years of age (67%) and qualified nurses (79%). Most (87%) reported having no formal mental health qualification, and 91% reported not undertaking any mental health continuing professional development during the previous five years of practice. Almost two thirds (59%) had worked in their professional role for over 5 years.

**Table 1:** Social and demographic characteristics of participants (n = 212)

Variable	Total	
	n	(%)
Sex		
Male	51	24.4
Female	158	75.6
Age-group		
< 30 years	52	24.9
30–39 years	87	41.6
40–49 years	41	19.6
> 50 years	29	13.9
Marital status		
Single	43	20.8
Married	155	74.9
Widowed	8	3.9
Separated/divorced	1	0.5
Highest level of education		
Diploma/certificate	190	90.9
Degree	19	9.1
Professional role in the primary health care setting		
Registered nurse	123	60.0
Enrolled nurse	39	19.0
Clinical officer	37	18.0
Medical officer	2	1.0
Other^a^	4	2.0
Formal mental health qualifications		
Yes	27	13.0
No	181	87.0
Continuing professional development in mental health in past 5 years		
Yes	18	8.7
No	190	91.3
Post-qualification experience		
≥ 5 years	120	58.8
< 5 years	84	41.2

^a^Other included public health technicians and community health workers in primary health care services

### Knowledge of mental health symptoms and diagnosis

Of the 212 completed questionnaires, 104 (49.1%) were related to the vignette on depression and 108 (50.9%) were related to the vignette on schizophrenia.

Although the majority of participants (65%) were able to identify that the symptoms described in their specific vignette were related to a mental health problem (‘has a problem’, ‘brain/mind problem’, ‘mental illness’), only a few were able to identify the diagnosis, i.e. depression or schizophrenia.

Participants diagnosed depression more frequently than schizophrenia (p = 0.034). Only 39% of the 104 participants who considered the depression vignette correctly identified the disorder; even fewer participants who considered the schizophrenia vignette could identify the disorder: 24% of 108 participants.

Table 2 shows diagnostic accuracy by level of education, professional role, formal mental health qualification, continuing professional development in the past 5 years and years of professional experience. None of these factors was significantly associated with diagnostic accuracy.

**Table 2:** Associations between diagnostic accuracy and social and demographic factors (n = 212)

Variable	Accurate diagnosis		p-value
	Yes n (%)	No n (%)	
Education			
Diploma/certificate	59 (31.1)	131 (68.9)	1.000
Degree	6 (31.6)	13 (68.4)	
Profession			
Registered nurse	42 (34.1)	81 (65.9)	0.493
Enrolled nurse	12 (30.8)	27 (69.2)	
Clinical officer	8 (21.6)	29 (78.4)	
Formal mental health qualification			
Yes	9 (33.3)	18 (66.7)	0.978
No	56 (30.9)	125 (69.1)	
CPD^a^			
Yes	3 (16.7)	15 (83.3)	0.258
No	62 (32.6)	128 (67.4)	
Post-qualification experience			
≥ 5yrs	41 (34.2)	79 (65.8)	0.289
< 5yrs	22 (26.2)	62 (73.8)	

^a^CPD (Continuing professional development in the previous 5 years)

### Helpful care strategies for a person with mental illness

This item required participants to consider medical, pharmacological and social interventions that would be considered for a person with symptoms described in their allocated vignette. The questionnaire item read:

### How do you think the person described in the vignette can be helped? Choose all that apply from this list

With regard to strategies and people who could be helpful to support a person with the symptoms described in the vignette, Table 3 presents a summary of frequencies and confidence intervals (95% CI) in ranked order from most commonly selected [1], to the least commonly selected [12].

**Table 3:** Ranked frequencies and confidence intervals of interventions by helpfulness (n = 212^a^)

Rank order	Intervention^a^	N (%)	95%CI
1	See a psychiatrist	67 (31.8)	25.6–38.4
2	Take to a mental hospital	29 (13.7)	8.5–18.5
3	Talk with friends and family	25 (11.8)	7.6–16.1
4	Listen and try to understand the problem	21 (10.0)	5.7–14.7
5	See a doctor	18 (8.5)	4.7–12.8
6	Take medication	10 (4.7)	1.9–8.0
7	Give love and affection	9 (4.3)	1.9–7.6
8	Patient must first recognise the problem	8 (3.8)	1.4–6.6
9	Don’t know	8 (3.8)	1.4–6.6
10	See a clinical officer	7 (3.3)	1.4–6.2
11	Make happy and encourage him/her	6 (2.8)	0.9–5.2
12	See/take to faith/traditional healer	3 (1.4)	0.0–2.8

^a^Multiple responses were recorded

Over half the participants (62%, n = 131), viewed medical interventions and/or seeing a medical professional (doctor, psychiatrist, nurse and/or medical social worker) as helpful for a person with mental illness. Social interventions such as ‘talk with family and friends’, ‘listen and try to understand the problem’, ‘give love and affection’ and ‘make happy and encourage him/her’ were considered favourably by 32.7% (n = 61) of participants. Clinical officers were considered helpful by only 3.3% (n = 7) of participants. Religious/faith and traditional healers were considered helpful by the lowest number of participants with only 1.4% (n = 3) of participants ranking this option as number 1.

### Attitudes towards people with mental illness

To gauge participants’ attitudes towards the person in their allocated vignette, a range of statements was presented. Participants were asked to indicate the likelihood of the person in the vignette to: be violent, have poor relationships, attempt suicide etc. or to have a good marriage, be a good parent or understand other people’s feelings.

Participants’ responses to positive and negatively worded statements were similar and did not differ by vignette type, and were therefore collapsed during analysis as shown in Table 4.

**Table 4:** Frequency distribution of participant responses to attitude statements (n = 201)

Attitude statement: People like this are likely to…	Statement implication	Likely n (%)	Don’t know n (%)	Unlikely n (%)
Be violent	Negative	15 (7.8)	4 (2.1)	173 (90.1)
Have poor relationships	Negative	28 (14.7)	4 (2.1)	158 (83.2)
Attempt suicide	Negative	30 (15.8)	4 (2.1)	156 (82.1)
Have understanding of other people’s feelings	Positive	157 (83.5)	9 (4.8)	22 (11.7)
Have a good marriage	Positive	169 (89.9)	7 (3.7)	12 (6.4)
Be a good parent	Positive	173 (92.0)	8 (4.3)	7 (3.7)
Be collegial	Positive	176 (93.1)	6 (3.2)	7 (3.7)

The majority of participants reported favourable perceptions of people with symptoms similar to those described in their clinical vignette, with high ratings for good parenting (92%), good marriage (90%) and ability to understand other people’s feelings (empathy) (84%). Very few participants considered the person described in their vignette as violent (8%), associated with poor interpersonal relationships (15%), or suicide attempts (16%).

### Attitudes towards socialising with a person with mental illness

A summary of the proportion of participants who agreed with each of 12 statements reflecting attitudes towards people with mental illness is presented in Table 5.

**Table 5:** Participant responses to positively and negatively worded statements

Attitude statements	Agree	
	n (%)	95%CI
Positive statements: I would…		
Agree to socialise with this person?	138 (83.1)	77.1–88.6
Accept as a team member at work	125 (75.3)	68.1–81.9
Agree to be a neighbour	120 (72.3)	65.7–78.9
Accept relationship by marriage	118 (71.1)	63.9–78.3
Accept interpersonal relationship	102 (61.4)	53.6–69.3
Would vote for the person	26 (15.7)	10.2–21.1
Negative statements		
Sign of personal weakness	9 (5.4)	2.4–9.0
Best to avoid this person	11 (6.6)	3.0–10.8
Not a real medical problem	18 (10.8)	6.6–16.2
People with this illness are erratic	75 (45.2)	37.3–52.4
Should be able to snap out of it	106 (63.9)	56.6–71.1
People with this illness are dangerous	89 (53.6)	45.8–62.0

With regard to the six positively worded statements, most participants agreed that they would associate these with a person with mental illness, in social, work, neighbourhood and personal relationships. Only a few (15.7%, n = 26) agreed with willingness to vote for a person with mental illness. Despite overwhelmingly positive perceptions of people with mental illness, 63.9% (n = 106) of participants indicated that a person with mental illness could ‘snap out of it’.

## Discussion

This study revealed that primary health care workers had very low mental health literacy indicated by low diagnostic accuracy for serious and common mental disorders; only 39% of participants allocated the depression vignette correctly identified the disorder, and diagnostic accuracy was even lower for schizophrenia (24%). Low diagnostic accuracy levels can have significant implications for treatment and care that people accessing health services receive. Antidepressant medications and antipsychotic medications are included in the Kenya Essential Package for Health [20]; this implies that nurses and clinical officers working in primary health care services are expected to prescribe and/or administer these medications when a diagnosis of schizophrenia or depression is made. While this study did not explore participants’ clinical practice treatment choices in response to symptoms in their allocated vignette, the results imply that most people presenting with mental health symptoms in primary health care services in Kenya may not receive accurate diagnosis and/or treatment.

According to WHO’s mhGAP, transforming the health workforce does not only involve training more health workers, but should also include building the capabilities of the existing workers [2, 7]. The primary health care system can be leveraged to enhance access to mental health services for people with mental illness where no specialist mental health services exist (Patel et al., 2016). Results from this study serve to highlight that primary health care workers in Kenya would require additional training for optimal mental health service provision, including assessment, diagnosis and treatment of people with mental illness. Marangu et al. have highlighted current capabilities within the Kenyan health workforce that can be leveraged in mental health capacity building (2014). Using the Capabilities Approach, the authors argued that high literacy levels, increased digitisation and harnessing the complementary role of traditional and faith healers can help to improve mental health care in Kenya [10].

The majority of participants in this study were women, under 40 years and with a diploma or certificate level of college education. These participant characteristics are consistent with other recent studies on the Kenyan health system that have reported a primarily young, female and college-educated health workforce [17, 25, 43]. This particular finding should be of interest to health planners because investments in capacity building initiatives such as training will be easier to scale up and sustain over a long time due to the relatively young age and current low literacy levels of the health workforce.

This study revealed that 87% of the participants did not have a formal mental health qualification, and 91% had not participated in any mental health specific continuing professional development in the past five years. These findings are consistent with other studies on the primary health care workforce undertaken in other LMICs such as India [44], Pakistan [38], South Africa [45] and Uganda [46]; these studies have reported low levels of formal mental health education, and/or continuing professional development among primary health care workers. In addition, WHO and the International Council for Nurses (ICN) [47] have highlighted the lack of adequate opportunities for education and training in mental health care globally. To meet this shortfall in training, mental health capacity-building programs require innovative approaches, such as the development of a diverse workforce of appropriately trained and supervised non-specialist health workers [48]. These authors have further singled out task-shifting as a potential solution for mental health care in LMICs that lack a specialist mental health workforce such as psychiatrists, psychologists and psychiatric/mental health nurses, whereby the service should be restructured by moving specific tasks away from professionals with higher qualifications to those with fewer or lower qualifications [48].

Nurses and clinical officers provide the bulk of mental health care services in Kenya in both hospital and primary care settings. There are very few doctors and even fewer psychiatrists, especially in rural areas [49]. Despite this, when participants in this study were asked to identify helpful persons to whom they would refer the person with mental illness described in the vignette, psychiatrists were the most endorsed option (32%), while only 3% of participants considered clinical officers helpful, despite their prominent role in primary health care services. This finding is interesting given that survey participants were health care workers, who did not seem to recognise their own role in mental health care provision, but rather ascribed mental health care as a tertiary function more suited for psychiatrists. Re-orienting the primary health care workforce on their role in mental health care would be key to mental health capacity building in Kenya.

Overall, participants’ responses to the questionnaire items on attitudes towards people with mental illness highlighted positive attitudes, with the majority indicating that people with mental illness were capable of being good parents, maintain a successful marriage and have empathy towards other people. These findings of positive perceptions are consistent with the African community care ethos described by Noor et al., that despite long distances and failing health systems, community ethos ensures families and communities take care of the sick and the vulnerable until appropriate care is available [50]. Despite these positive perceptions, 64% of participants responded that the person described in the vignette could ‘snap out of it’, despite the respondents being college-trained health care workers. This is indicative of low mental health literacy levels in the health workforce. Positive perceptions about people with mental illness are important in education and campaigns designed to combat stigma against mental illness [51]. These findings of positive perceptions in Kenya can be leveraged when planning and implementing mental health capacity building programs and human rights awareness campaigns for primary health care workers.

### Implications for health policy and practice

Kenya has aligned itself with other countries to achieve the United Nation’s Sustainable Development Goals (SDGs), including attainment of SDG-3 (Good Health) [14, 33]. Through the national development agenda Vision 2030, Kenya aims to transform itself into a globally competitive, prosperous and industrialising middle-income country [13]. Furthermore, the Kenyan 2010 constitution has proclaimed a comprehensive, people-driven and rights-based approach to health service provision [52]. To achieve these goals, good and accessible health care, including mental health care, for all the population is essential. Recent initiatives such as launching of the national mental health policy in 2015 [53], and commissioning of the national mental health taskforce by the president of Kenya are good developments and steps in the right direction.

Findings from this study provide new insights into current gaps relating to mental health literacy levels of the primary health care workforce in Kenya and similar countries.

## Limitations

Jorm’s MHLQ [28] has been widely used to undertake mental health literacy surveys of health care workers and the general public in many settings including in LMICs; however, while the instrument was adapted for use in Kenya, it was not validated for the Kenyan context. Our findings may be further limited by the self-report approach used to collect data from participants. Participants’ self-reported mental health literacy may differ from their actual clinical practice.

Further potential limitation of this study was the low response rate. The authors acknowledge that the response rate was low at 25%. Although such low response rates are not uncommon in unsolicited surveys, especially among busy health care workers, this implies that results should be interpreted with caution as they may not be representative of the workforce.

## Conclusions

This study provides insights into mental health literacy of the primary health care workforce in Kenya. The study has highlighted the need for interventions that can help to develop and enhance mental health knowledge and skills among primary health care workers to ensure adequate and effective response to the disease burden related to mental illness in Kenya. The most salient finding was the very low levels of diagnostic accuracy for mental illness among participants whose primary role includes assessment, diagnosis and treatment of mental illness. Similarly, the finding that psychiatrists were frequently endorsed as the most helpful source of support for a person with mental illness was concerning considering their very low numbers in the country.

To our knowledge, the current study is the first to report on mental health literacy of the primary health care workforce in Kenya. Results from the study provide an important empirical baseline from which future mental health and educational interventions can be planned. This research also helps to address the dearth of research into the capacity of primary health care workers who provide the bulk of health care in Kenya and similar countries.

## Data Availability

The data that support the findings of this study are available from Deakin University Server, but restrictions apply to the availability of these data as per current Deakin University research data storage and retrieval policies. These data are not publicly available, however with advance notice, the corresponding author (EM) can make whole data set (or part of) available subject to permission of Deakin University.

## Abbreviations

ICN: International Council of Nurses
KMHLQ: Kenya Mental Health Literacy Questionnaire
LAMIC: Low and middle-income countries
mhGAP: Mental Health Global Action Program
MHL: Mental health literacy
MHLQ: Mental Health Literacy Questionnaire
SDGs: Sustainable Development Goals
WHO: World Health Organization
WHO-AIMS: World Health Organization Assessment Instrument for Mental Health Systems
